# A case report of pseudoaneurysm of left sinus of Valsalva invaded into the left ventricle with severe aortic regurgitation

**DOI:** 10.1186/s13019-018-0754-1

**Published:** 2018-06-07

**Authors:** Hyun Oh Park, Joung Hun Byun, Seong Ho Moon, Jong Woo Kim, Sung Hwan Kim, Ki Nyun Kim, Jae Jun Jung, Dong Hoon Kang, Jun Young Choi, Jun Ho Yang, In Seok Jang, Chung Eun Lee

**Affiliations:** 1 Department of Thoracic and Cardiovascular Surgery, Changwon Hospital, Gyeongsang National University College of Medicine, 11, Samjeongja-ro, Seongsan-gu, Changwon-si, 51472 Republic of Korea; 2 Department of Thoracic and Cardiovascular Surgery, Jinju Hospital, Gyeongsang National University College of Medicine, Jinju, Republic of Korea

**Keywords:** Aneurysm, Rupture, Sinus of Valsalva2

## Abstract

**Background:**

The pseudoaneurysms of sinus of Valsalva is an uncommon and serious complication of an infection, trauma, or after cardiac surgery or procedure. Pseudoaneurysms of sinus of Valsalva from left is rare. We describe a case of pseudoaneurysm of the left coronary sinus of Valsalva invaded into the left ventricle (LV) diagnosed by transthoracic echocardiography (TTE), transesophageal ecoccardiography (TEE), and multiple detector computed tomography (MDCT).

**Case presentation:**

A 44-year-old male patient had New York Heart Association (NYHA) class II / III dyspnea during 4 months. He underwent surgery including aortic valve replacement using mechanical prosthesis, and he was discharged well without significant complications on follow – up TTE and chest computed tomography (CT) post-operative 7 days.

**Conclusions:**

We report this rare case in which a ruptured pseudoaneurysm of sinus of Valsalva into LV with severe AR due to perforation of LCC was successfully-treated.

## Background

The pseudoaneurysms of sinus of Valsalva is an uncommon and serious complication of an infection, trauma, or after cardiac surgery or procedure [1]. In most cases, it arise from right and non-coronary sinuses, and pseudoaneurysms of sinus of Valsalva from left is rare. It can form aortocavitary fistulae with the adjacent cardiac chamber [2]. We report a case of patient who underwent surgical collection for the pseudoaneurysms of sinus of Valsalva invaded into the LV combined with left coronary cusp (LCC) perforation occurring without previous any procedures, heart surgery and definitive infection signs.

## Case presentation

**Fig. 1:**
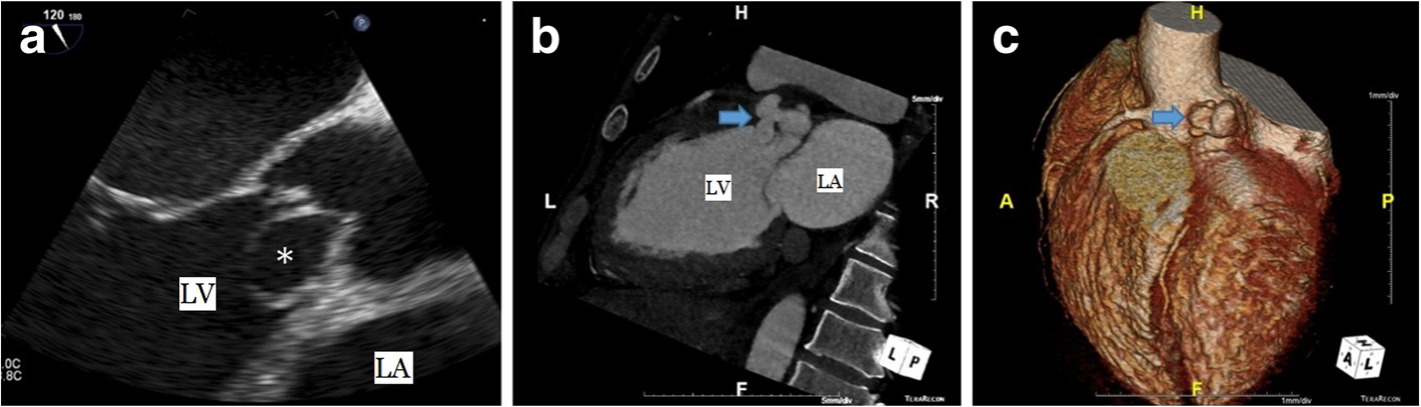
**a** Transthoracic echocardiography shows the sac in the left ventricle (asterisk). **b**, **c** Multidetector computed tomography shows a ruptured pseudoaneurysm of sinus of Valsalva originated from left coronary sinus into left ventricle (arrow). LV, left ventricle; LA, left atrium

A 44-year–old male was referred to our hospital with diagnosis of severe aortic regurgitation (AR). He had New York Heart Association class II/III dyspnea on exertion for last 4 months. He had no another clinical symptoms, including prolonged fever or chilling sensation. He had a history of pulmonary tuberculosis (TB) on medication 12 weeks previously, but no history of chest trauma, hypertension, diabetes, and connective tissue disorder. Cardiovascular examinations was suggestive of single S2 and grade I*V*/V diastolic murmur at aortic area. On examination, his chest X-ray revealed cardiomegaly and electrocardiogram was normal sinus rhythms. However, preoperative TTE and TEE revealed global hypokinesia with moderate left ventricle (LV) dysfunction (ejection fraction: 40%), severe eccentric AR suspected of being caused by LCC perforation, and aneurysmal sac in LV (Fig. 1a). MDCT revealed rupture of pseudoaneurysm originated from left coromary sinus (LCS) into LV (Fig. 1b, c).

We planned to carry out surgical correction after sufficient treatment for the heart failure and achieving sputum culture negative conversion. We could not reliably distinguish between aortic valve pathology and subaortic aneurysmal sac outpouching into LV before surgery.

**Fig. 2:**
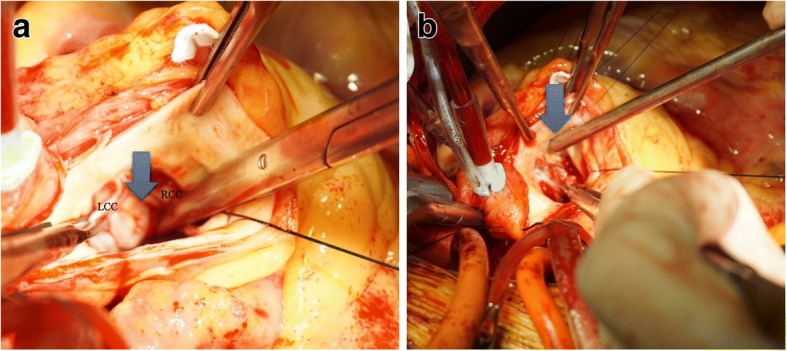
**a** Operative filed shows the sac in the left ventricle. It originated from near left coronary cusp (arrow). **b** The suction tip penetrates the opening for pseudoaneurysm (arrow). LCC, left coronary cusp; RCC, right coronary cusp

**Fig. 3:**
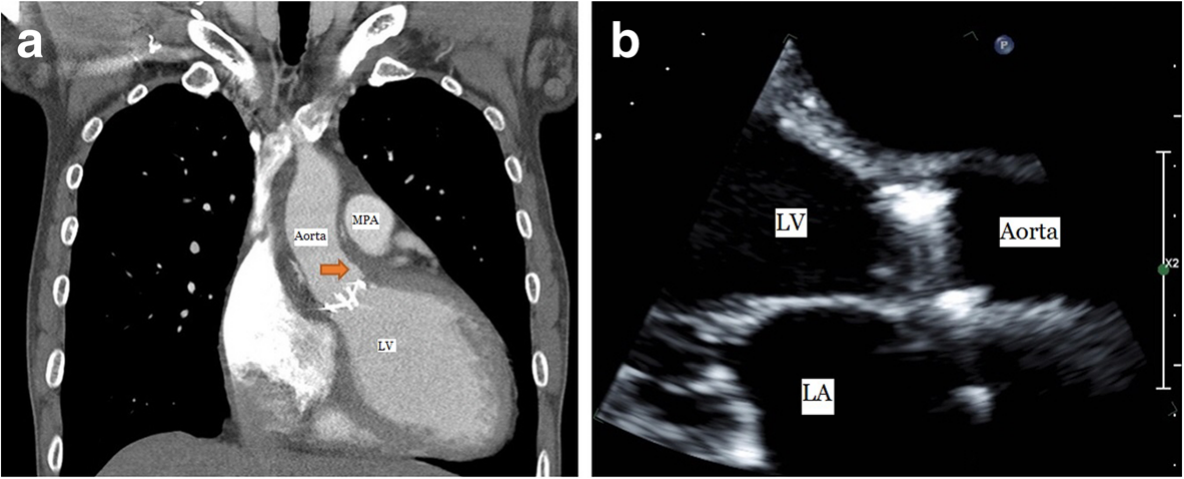
**a** Postoperative chest computed tomography shows that pseudoaneurysm of sinus of Valsalva is only a trace left (arrow). **b** Transthoracic echocardiography shows that the sac in left ventricle disappeared. MPA, main pulmonary artery; LV, left ventricle; LA, left atrium

At surgery, routine cardiopulmonary bypass (CPB) including administration of antegrade and retrograde cardioplegia solution for cardiac arrest was performed. After standard oblique aortotomy, we found perforation of LCC, ruptured subaortic aneurysmal sac outpouching into LV, and orifice lacked intima tissue for pseudoaneurysm of LCS (Fig. 2). The tissue near the LCC perforation and orifice for pseudoaneurysm of LCC showed fibrotic changes. There was almost no valve tissue remaining in the aortic annulus of the LCC perforated area. The orifice for pseudoaneurysm of LCS was obliterated by separating commissure between the LCC and right coronary cusp (RCC), and suturing twice with 4–0 plolene using the commissural tissue because there was not enough surrounding tissue for patchy closure. Since the subaortic aneurysmal sac structure was sufficiently strong after removing a part of the sac, the orifice of the LV side was obliterated using a part of the sac structure and autologous pericardial patchy. And then aortic valve replacement using a 21 mm mechanical prosthesis (ST. JUDE MEDICAL Inc., Minnesota, U S A) was performed in routine fashion. We could wean CPB without significant complications. He had an uneventful course and the follow-up TTE after 7 days of surgery showed the well function of prosthetic valve with moderate LV dysfunction and no remnant aneurysmal sac. Postoperative chest CT (Fig. 3a) and TTE (Fig. 3b) showed no pseudoaneurysm of sinus of Valsalva. Histologically, no evidence of infection, inflammation, and TB was seen. The patient’s post-operative hospital course was uneventful without any complications. He was followed up for 5 months after discharge.

## Discussion and conclusion

True aneurysms of sinus of Valsalva are either congenital or acquired presenting infrequent occurrence. Congenital true aneurysms of sinus of Valsalva are caused by a defect in the medial layer of the wall of the aortic sinus [3]. Uncommonly, acquired true aneurysm of sinus of Valsalva can be associated with infection (e.g. endocarditis, syphilis and tuberculosis), degenerative disease (e.g. atherosclerosis and connective tissue disorder) or chest trauma. It can rupture into adjacent cardiac chambers, those on right side are more affected. Rarely, they ruptured into left – sided heart, the pulmonary artery or pericardial space [1, 3].

Pseudoaneurysm of sinus of Valsalva are results of blood collecting within a false lumen after intimal tear due to trauma caused by catheter procedures or surgeries [1]. Rarely, infective endocarditis (IE) may be etiology of pseudoaneurysm of sinus of Valsalva. IE can cause the paravalvular abscess or valvular insufficiency. Especially, pseudoaneurysm of sinus of Valsalva associated with paravalvular abscess is estimated to occur in 28% in IE cases [2, 4]. Pseudoaneurysm of sinus of Valsalva most often involve the right coronary sinus (RCS) (80%) and the noncoronary sinus (NCS) (16%), and the LCS (4%) [5].

Our patient had no other symptoms besides dyspnea and no definitive evidence of IE including negative blood culture and excised aortic valve (AV) tissue culture. Assuming a perforation of AV, it may be presumed that there was a previous asymptomatic infection.

Preoperative diagnosis for both true aneurysm and pseudoaneurysm can be possible by TTE, TEE, CT angiography, magnetic resonance imaging (MRI) and catheterization. In our case, MDCT was helpful in identifying the anatomic relationship between pseudoaneurysm and aortic root. At imaging, the criteria for diagnosing an aneurysm of sinus of Valsalva include an origin above the aortic annulus, a saccular shape, and normal dimension of the adjacent aortic root and ascending aorta. TTE initially visualized an aneurysm of sinus of Valsalva; it most patients, angiography is considered the reference standard for confirming the presence of an aneurysm of sinus of Valsalva and any aortocardiac shunt. MRI imaging also allows accurate assessment of the origin, size, and rupture of aneurysm of sinus of Valsalva and the status of the surrounding anatomy. Additionally, the advantage of MRI include the ability to evaluate the left ventricular hemodynamic pattern, identify AR [6].

In our case, CT was the most helpful in the diagnosis of pseudoaneurysm. CT was read by three experienced radiologists and two cardiac surgeons, and there was no connection of the aortic intima to the entrance of sac in the operative field.

We report this rare case in which a ruptured pseudoaneurysm of sinus of Valsalva into LV with severe AR due to perforation of LCC was successfully-treated. In our patient, it may be presumed that the patient had a chronic inflammatory pseudoaneurysm due to tuberculosis or any other 2nd infection, according to clinical course, radiologic and operative filed findings (continuity with the intimal fissure of the sinus of Valsalva). Conclusion: This report is important because even though tuberculosis was not confirmed histologically and it deserves to be considered as a rare cause, it draws attention to the possibility of variant causes of an aneurysm of sinus of Valsalva.

## Abbreviations

CPB: Cardiopulmonary bypass
LCC: Left coronary cusp
LCS: Left coromary sinus
LV: Left ventricle
MDCT: Multiple detector computed tomography
NCS: Noncoronary sinus
RCS: Right coronary sinus
TB: Pulmonary tuberculosis
TEE: Transesophageal ecoccardiography
TTE: Transthoracic echocardiography
